# A Pressure-Based Multiphase Flowmeter: Proof of Concept

**DOI:** 10.3390/s23167267

**Published:** 2023-08-19

**Authors:** Vijay Ramakrishnan, Muhammad Arsalan

**Affiliations:** 1 Aramco Americas, Houston, TX 77084, USA; 2 Saudi Aramco, Dhahran 31311, Saudi Arabia

**Keywords:** densitometer, flowmeter, multiphase, pressure

## Abstract

Multiphase flowmeters (MPFMs) measure the flow rates of oil, gas, and brine in a pipeline. MPFMs provide remote access to real-time well production data that are essential for efficient oil field operations. Most MPFMs are complex systems requiring frequent maintenance. An MPFM that is operationally simple and accurate is highly sought after in the energy industry. This paper describes an MPFM that uses only pressure sensors to measure gas and liquid flow rates. The design is an integration of a previously developed densitometer with an innovative Venturi-type flowmeter. New computing models with strong analytical foundations were developed, aided by empirical correlations and machine-learning-based flow-regime identification. A prototype was experimentally validated in a multiphase flow loop over a wide range of field-like conditions. The accuracy of the MPFM was compared to that of other multiphase metering techniques from similar studies. The results point to a robust, practical MPFM.

## 1. Introduction

A multiphase flowmeter (MPFM) measures the volumetric flow rates of multiple fluids/phases flowing through a pipeline. An MPFM is typically installed between the wellhead and the separator in an oil/gas production field. MPFMs are widely used in the energy industry for production optimization and reservoir management [1]. 

The operators use data from the MPFM for various purposes [1,2,3]. During routine operations, the data help to ensure that oil/gas production rates are within target ranges based on the capacity of downstream processing facilities. In case of an upset condition, the data are beneficial for timely corrective actions such as choke adjustment. In artificial-lift operations, these data help to optimize operational parameters such as the lift-gas injection rate or pump speed. For reservoir management, MPFM data represent a valuable input for maximizing long-term oil/gas extraction.

A typical MPFM [2] primarily measures the total mass flow rate, mixture density, and water liquid ratio, from which it calculates the oil, gas, and brine flow rates. To make these three primary measurements, MPFMs use various combinations of sensors, at least three, working in tandem [2,3]. One of the biggest challenges for these sensors is that multiphase flows are highly unsteady and non-homogenous. For example, a multiphase flow can be in one of several flow regimes (such as ‘bubbly’, ‘churn’, ‘slug’, or ‘annular’) or it may transition continuously (and frequently) between those regimes [2]. Another major challenge is that gas and liquid phases typically travel at different velocities—a phenomenon called slip [4,5]—that can bias the raw sensor response. Many multiphase sensors are also sensitive to fluid properties (such as salinity and viscosity). To deal with these challenging flow conditions, most multiphase sensors require special calculation models, periodic maintenance, and frequent tuning.

Table 1 summarizes common measurement principles for the total mass flow rate and fluid mixture density. For measuring the total flow rate, differential pressure devices are very common ([6,7,8]). These devices are based on Bernoulli’s principle (ca. 1738); non-ideal losses are empirically characterized using a ‘discharge coefficient’ (*C_d_*). While *C_d_* correlations are well-understood in single-phase flows [9], they are more involved in multiphase flows [10]. For measuring mixture density, gamma-ray attenuation is one of the most prevalent techniques in oil-and-gas applications ([6,7,8]). Another well-known technique for measuring the total mass flow rate is the Coriolis-force-induced deformation/twisting of a vibrating flow tube [11]. A ‘Coriolis meter’ can additionally measure fluid density based on the natural frequency of tube vibration. Another multiphase metering technique is the use of a strain–sensor array that measures the speeds of propagating pressures in the fluid stream (i.e., both the fluid velocity and sound speed) [12]. It is a ‘full bore’ measurement because it has no intrusive components or flow constrictions. While all the above are ‘inline’ techniques that do not physically separate the phases, another class of MPFMs uses a built-in separator. These systems measure each phase separately using single-phase flow meters that are installed downstream of the separator [13]. 

All the above-mentioned measurement techniques have unique limitations. The gamma-ray technique ([6,7,8]) uses nuclear sources which have safety concerns and logistical hassles. A Coriolis meter may underperform at high gas fractions (above 20%), particularly with respect to measuring density, due to the phenomenon of gas–liquid decoupling [11]. Strain–sensor arrays may underperform at medium gas fractions (in the 20–80% range) due to a fundamentally weak relationship between the sound speed and fluid density [12]. Separation-based systems are larger/heavier, have smaller rangeability, and rely on the frequent adjustment of valves to control flow separation [13].

This article describes a new MPFM that uses only differential pressure sensors to measure both the total flow rate and mixture density. Differential pressure sensors are mature technologies that are easy to maintain and reliable, even in harsh environments. This translates to lower operational costs, especially for remote installations that are common in oil-and-gas applications. Furthermore, the new MPFM design leverages conventional piping arrangements without any special/custom machining, thus minimizing manufacturing costs. This article comprehensively explores whether this favorable sensing technique can be innovatively harnessed to accurately measure gas and liquid rates under all multiphase flow conditions.

## 2. Instrument Design

### 2.1. Concept and Design

**Figure 1:**
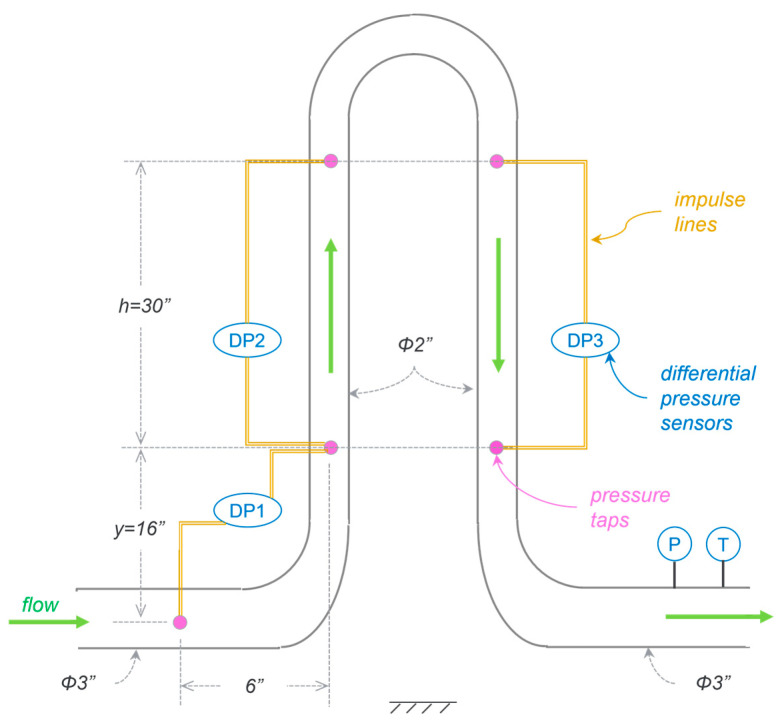
Concept and design of a multiphase flowmeter that uses just pressure measurements.

The MPFM has six pressure taps instrumented with differential pressure sensors (*DP*_1_, *DP*_2_, and *DP*_3_) as shown in Figure 1. The horizontal inlet pipe is 3″ in diameter, and the two vertical pipes are 2″ in diameter. *DP*_1_ is measured across a reducing elbow, as shown below. *DP*_2_ and *DP*_3_ are measured across an equal height (*h*) in the upward and downward flow columns, respectively. 

As described in [14], *DP*_2_ is the sum of a downward hydrostatic (gravitational) force and a downward (frictional) drag force, while *DP*_3_ is the sum of a downward hydrostatic (gravitational) force and an upward (frictional) drag force. For a given flow/fluid condition, the frictional force will ideally be equal and opposite. So, the term *DP*_2_ + *DP*_3_ cancels out the frictional losses, yielding the total hydrostatic head, i.e.,

(1) $$DP_{2}+DP_{3}=(\rho_{m}gh)\cdot 2$$

where $\rho_{m}$ is the mixture density. From Equation (1), $\rho_{m}$ can be readily calculated using

(2) $$\rho_{m}=\left(DP_{2}+DP_{3}\right)/\left(2gh\right)$$

Due to the change in the flow’s cross-sectional area from a 3″ pipe to a 2″ pipe, *DP*_1_ is a strong function of total mass flow rate (${\dot{m}}_{t}$), akin to a classical Venturi meter [9]. But, to account for the downstream tap location at a height of ‘*y*’ and the resultant hydrostatic head, the standard equation for ${\dot{m}}_{t}$ must include a correction term ($\rho_{m}gy$) such that

(3) $${\dot{m}}_{t}=KC_{d}\sqrt{2\left(DP_{1}-\rho_{m}gy\right)\rho_{m}}$$

Thus, the MPFM measures both the mixture density and the mass flow rate by strategically placing pressure sensors in a conventional piping arrangement.

### 2.2. Calculation Model

**Figure 2:**
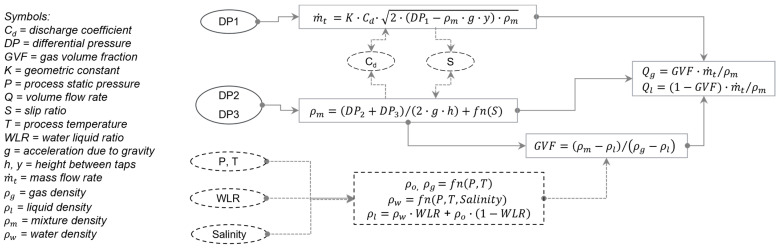
Flow chart of the calculation model—from raw pressure measurements to phase flow rates.

For the above flow-metering concept, a calculation model was developed to convert its primary measurements (mixture density and mass flow rate) into gas and liquid rates, as described in the flowchart in Figure 2. The model has seven inputs—three differential pressures (*DP*_1_, *DP*_2_, *DP*_3_), pressure (*P*), temperature (*T*), water liquid ratio (*WLR*), and salinity. The *WLR* is typically measured using a water-cut meter [12] or from a sample drawn from the pipeline [2]. For this study, the *WLR* was obtained from the test facility’s reference meters (described in the next section). Salinity is typically measured using a dedicated sensor [6] or obtained from a water sample drawn from the pipeline [2]. For this study, the latter technique was used. 

In this model, the gas and liquid densities were first calculated based on known pure-phase properties [15], using *P*, *T*, *WLR*, and salinity. The mixture density and mass flow rate were calculated next, using Equations (2) and (3). The discharge coefficient (*C_d_*) and slip ratio (*S*) were then iteratively calculated from empirical correlations [4,10]. Note that the mixture density and mass flow rate were also iteratively updated during this step. Then, the gas volume fraction (*GVF*) was calculated using known equations [2]. Finally, the phase flow rates (*Q*) at line conditions were calculated [2]. These flow rates can be subsequently converted (not shown) from line to standard conditions, if needed, based on the fluid composition and thermodynamic phase properties [15].

## 3. Experimental Setup and Test Envelope

To experimentally validate the concept (and the model), a prototype was tested at the Southwest Research Institute (SwRI)’s multiphase flow test facility in San Antonio, Texas, USA. The flow loop had a multiphase pump and a three-phase separator instrumented with Coriolis meters in the oil/water legs and the orifice plate in the gas leg. The facility was able to pressurize the flow loop to a target pressure and circulate known flow rates of oil, brine, and gas through the test section in a closed loop. The SwRI test facility attests that the uncertainty of their reference data (for non-slugging, steady-state conditions) is as follows: liquid flow rates: <±1%; gas flow rates: <±1.5% (for low-flow conditions), and <±0.6% (for high-flow conditions).

**Figure 3:**
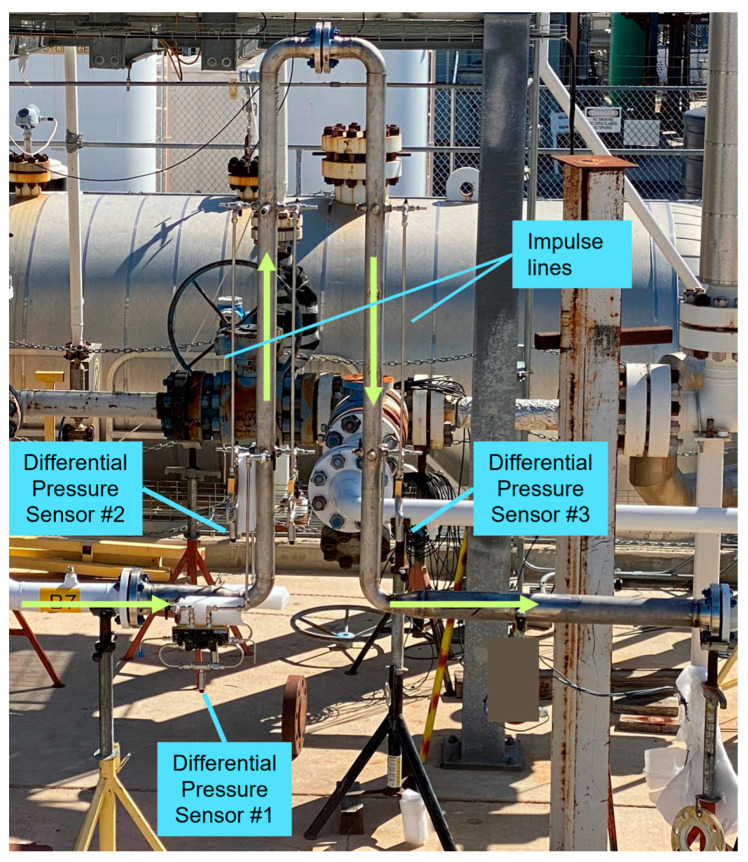
Picture of a prototype of the multiphase flowmeter installed in the test section of a flow loop.

The picture in Figure 3 shows the prototype installed in the test section of the flow loop. The pressure sensors had an accuracy of ±0.04% of full scale. Good practice guidelines [16] were followed in the design and operation of these sensors and impulse lines.

**Figure 4:**
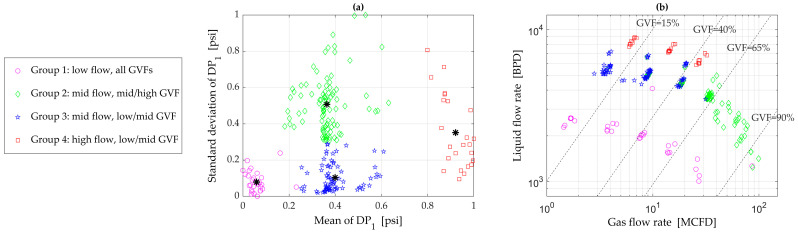
(**a**) Output from a k-means machine learning algorithm showing well-defined groups; (**b**) a flow map confirming that the groups indeed correlated well with different flow conditions.

The test envelope was selected to represent a typical oil well with a 3-inch production line. The range of flow conditions is summarized in Table 2. Within this test envelope, a total of 221 test points were chosen such that they included low, mid, and high flow levels, roughly equivalent to 2000, 5000, and 8000 BPD, respectively. The test points were distributed across the entire *GVF* range of interest. Figure 4b shows all test points in terms of the gas/liquid flow rates and *GVF*s. The test used methane, synthetic oil, and brine as the fluid media. Roughly half of the test points were at 4% salinity, and the remaining were at 13% salinity. The entire test was carried out at a nominal pressure of 200 psig and a temperature of 80 F.

Each test point was held statistically stationary for 5 min. During this time, the ‘actual’ flow rates from the test facility and the ‘measured’ flow rates from the MPFM were recorded and averaged.

## 4. Results and Discussion

### 4.1. Flow Regime Identification

As indicated earlier, to achieve the best accuracy from a differential pressure device with multiphase flows [10], its discharge coefficient (*C_d_*) must be well characterized based on flow conditions, and this necessitates an identification of the flow regime using raw data. The same logic can be extended to the slip ratio (*S*) which is also influenced by the flow regime [2]. In addition to improving the model’s accuracy, the flow-regime-identification step will also improve the model’s generalizability.

As a first step, features that correlate well with flow regimes were selected. The mean and standard deviation of *DP*_1_ are good predictors of the flow regime [17]. The mean of *DP*_1_ is a logical choice because it correlates well with the total mass flow rate which, in turn, strongly influences the type of flow regime [2]. The standard deviation of *DP*_1_ is also a logical choice because some flow regimes, such as slug flows, are more unsteady than others (such as bubbly/annular flows) [2]. 

As the next step, the k-means machine learning algorithm [18] was applied to the entire dataset. The k-means algorithm is an unsupervised classification algorithm that clusters data with similar characteristics based on distance (e.g., Euclidean distance) in a feature space. The features (the mean and standard deviation of *DP*_1_) were first normalized in the range of 0 to 1. The algorithm was implemented with k = 4, and the results are shown in Figure 4a, which shows clearly segregated groups. When these groups are plotted on a flow map (Figure 4b), they clearly correlate well with different flow conditions. For example, in mid-range flows, the test points in a mid-to-high *GVF* range, presumably slug flows, are in group 2, while those in a low-to-mid *GVF* range, presumably bubbly or churn flows, are in group 3. 

Finally, one simple linear regression was used per group to correlate *C_d_* and *S*.

### 4.2. Measurement Accuracy

The accuracy of the MPFM was quantified by comparing its measurements to flow loop (i.e., actual) data for gas flow rate (*GFR*) and liquid flow rate (*LFR*). For each test point, the relative measurement deviations (*ε*) were calculated as follows:

(4) $$\varepsilon_{GFR}=\frac{{GFR}_{MPFM}-{GFR}_{ACTUAL}}{{GFR}_{ACTUAL}}$$

(5) $$\varepsilon_{LFR}=\frac{{LFR}_{MPFM}-{LFR}_{ACTUAL}}{{LFR}_{ACTUAL}}$$

**Figure 5:**
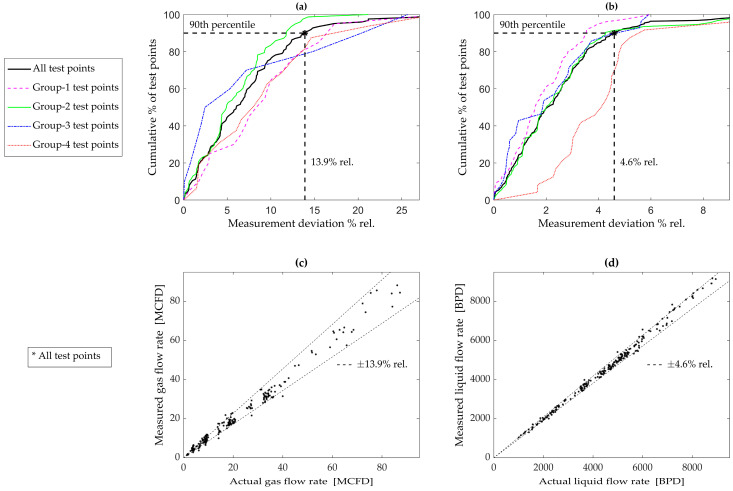
Empirical cumulative distribution of measurement deviations: (**a**) gas flow rate; (**b**) liquid flow rate. Measured vs. actual: (**c**) gas flow rate; (**d**) liquid flow rate.

The empirical cumulative distribution functions (eCDFs) of the absolute values of these deviations (*ε*) were plotted for all test points in Figure 5a (*GFR*) and Figure 5b (*LFR*). From Figure 5a,b, using the 90th percentile as the yardstick, the MPFM had an accuracy of ±13.9% (relative) for the *GFR* and ±4.6% (relative) for the *LFR*. Figure 5c,d are direct comparisons (of all test points) for the *GFR* and *LFR*, respectively.

### 4.3. Discussion

Figure 5a (*GFR*) and Figure 5b (*LFR*) also contain deviations specific to each of the four flow conditions/groups identified earlier. The graphs reveal that the liquid rate is least accurate under high-flow conditions (i.e., group 4 in Figure 5b). This is likely due to the *DP*_1_ pressure sensor occasionally exceeding its maximum, thus biasing the raw data. The gas rate is the least accurate for low-to-mid-range *GVF*s (i.e., groups 1, 3, and 4 in Figure 5a). This requires further investigation in future studies.

The overall accuracy of this technique compares well with other multiphase meters that were experimentally evaluated in multiphase flow loops in the published literature. Zhou et al. [19], using Coriolis techniques, attained an accuracy of 2.5% (97th percentile) for the *LFR* and 5% (93rd percentile) for the *GFR*. Hogendoorn et al. [20], using magnetic resonance techniques, attained an accuracy of around 5% (90th percentile) for the *LFR* and around 10% (90th percentile) for the *GFR*. The accuracy metrics of the new MPFM are generally on a par with other such instruments, thus validating its concept and design. But there is room for improvement, especially in the gas rate accuracy.

Future work will need to focus on two key aspects—accuracy and field readiness. The system’s accuracy can be improved by increasing the vertical separation between pressure taps, but the resultant taller system could be a problem for some applications if space is limited. The system can be made field-ready by using pressure sensors with remote diaphragm seals that prevent the fouling of impulse lines; however, diaphragm-seals degrade the accuracy of raw data from the sensors. The trade-offs noted above are also the limitations of this technology. In other words, future improvements will be a balancing act between accuracy, size, and ruggedness.

## 5. Conclusions

A non-nuclear multiphase flowmeter was realized using only pressure sensors. A prototype was experimentally validated in a multiphase flow loop, demonstrating good accuracy over a wide range of gas and liquid flow rates. Flow-regime identification was one of the key elements in the calculation model. The study determined that the largest measurement deviations occurred at gas fractions in the range of 25–50%. The next logical step is to optimize the pressure tap locations to improve the MPFM’s measurement accuracy. However, there is an inherent three-way tradeoff between accuracy, size, and field-readiness in the future roadmap of this technology. Regardless, given its simple, reliable design, this technique has the potential to be a compelling choice for inline multiphase metering in oil-and-gas applications.

## Figures and Tables

**Table 1:** Measurement principles used in select multiphase flow metering technologies.

Literature References	Measurement Principle for Total Flow Rate	Measurement Principle for Mixture Density
[6,7,8]	Differential pressure across area reduction	Gamma-ray attenuation
[11]	Coriolis (vibrating tubes): deformation	Coriolis (vibrating tubes): natural frequency
[12]	Pipe strain (cross correlation): flow velocity	Pipe strain (cross correlation): sound speed
[13]	Gas–liquid cyclonic separator, with a Coriolis meter in the liquid line and an orifice plate in the gas line	

**Table 2:** Test envelope for evaluating the multiphase flowmeter prototype.

Parameter	Range
Line pressure, *P*	~200 psig
Line temperature, *T*	~80 F
Liquid flow rate, *LFR*	1000–9000 BPD
Gas flow rate, *GFR*	0–90 MCFD
Gas volume fraction, *GVF*	6–93%
Water liquid ratio, *WLR*	0–100%

## Data Availability

The data that support the findings of this study cannot be made publicly available due to legal restrictions but are available upon reasonable request.
